# Practice transformations to optimize the delivery of HIV primary care in community healthcare settings in the United States: A program implementation study

**DOI:** 10.1371/journal.pmed.1003079

**Published:** 2020-03-26

**Authors:** Wayne T. Steward, Kimberly A. Koester, Mary A. Guzé, Valerie B. Kirby, Shannon M. Fuller, Mary E. Moran, Emma Wilde Botta, Stuart Gaffney, Corliss D. Heath, Steven Bromer, Starley B. Shade

**Affiliations:** 1 Center for AIDS Prevention Studies, Department of Medicine, University of California San Francisco (UCSF), San Francisco, California, United States of America; 2 U.S. Department of Health and Human Services (HHS), Health Resources and Services Administration (HRSA), HIV/AIDS Bureau, Rockville, Maryland, United States of America; 3 Department of Family and Community Medicine, University of California San Francisco (UCSF), San Francisco, California, United States of America; 4 Institute for Global Health Sciences, University of California San Francisco (UCSF), San Francisco, California, United States of America; Boston University School of Public Health, UNITED STATES

## Abstract

**Background:**

The United States HIV care workforce is shrinking, which could complicate service delivery to people living with HIV (PLWH). In this study, we examined the impact of practice transformations, defined as efficiencies in structures and delivery of care, on demonstration project sites within the Workforce Capacity Building Initiative, a Health Resources and Services Administration (HRSA) Ryan White HIV/AIDS Program Special Projects of National Significance (SPNS).

**Methods and findings:**

Data were collected at 14 demonstration project sites in 7 states and the District of Columbia. Organizational assessments were completed at sites once before and 4 times after implementation. They captured 3 transformation approaches: maximizing the HIV care workforce (efforts to increase the number of existing healthcare workforce members involved in the care of PLWH), share-the-care (team-based care giving more responsibility to midlevel providers and staff), and enhancing client engagement in primary HIV care to reduce emergency and inpatient care (e.g., care coordination). We also obtained Ryan White HIV/AIDS Program Services Reports (RSRs) from sites for calendar years (CYs) 2014–2016, corresponding to before, during, and after transformation. The RSR include data on client retention in HIV care, prescription of antiretroviral therapy (ART), and viral suppression. We used generalized estimating equation (GEE) models to analyze changes among sites implementing each practice transformation approach. The demonstration projects had a mean of 18.5 prescribing providers (SD = 23.5). They reported data on more than 13,500 clients per year (mean = 969/site, SD = 1,351). Demographic characteristics remained similar over time. In 2014, a majority of clients were male (71% versus 28% female and 0.2% transgender), with a mean age of 47 (interquartile range [IQR] 37–54). Racial/ethnic characteristics (48% African American, 31% Hispanic/Latino, 14% white) and HIV risk varied (31% men who have sex with men; 31% heterosexual men and women; 7% injection drug use). A substantial minority was on Medicaid (41%). Across sites, there was significant uptake in practices consistent with maximizing the HIV care workforce (18% increase, *p* < 0.001), share-the-care (25% increase, *p* < 0.001), and facilitating patient engagement in HIV primary care (13% increase, *p* < 0.001). There were also significant improvements over time in retention in HIV care (adjusted odds ratio [aOR] = 1.03; 95% confidence interval [CI] 1.02–1.04; *p* < 0.001), ART prescription levels (aOR = 1.01; 95% CI 1.00–1.01; *p* < 0.001), and viral suppression (aOR = 1.03; 95% CI 1.02–1.04; *p* < 0.001). All outcomes improved at sites that implemented transformations to maximize the HIV care workforce or improve client engagement. At sites that implemented share-the-care practices, only retention in care and viral suppression outcomes improved. Study limitations included use of demonstration project sites funded by the Ryan White HIV/AIDS Program (RWHAP), which tend to have better HIV outcomes than other US clinics; varying practice transformation designs; lack of a true control condition; and a potential Hawthorne effect because site teams were aware of the evaluation.

**Conclusions:**

In this study, we found that practice transformations are a potential strategy for addressing anticipated workforce challenges among those providing care to PLWH. They hold the promise of optimizing the use of personnel and ensuring the delivery of care to all in need while potentially enhancing HIV care continuum outcomes.

## Abstract 2

Wayne Steward and co-workers report on implementation of practice improvement projects for HIV care in the United States.

## Author summary

**Why was this study done?:**

- The study aimed to enhance the capacity and readiness of Ryan White HIV/AIDS Program (RWHAP)-funded clinics to adapt and realign their workforce and practices to improve the provision of quality HIV care.
- The study aimed to identify practice transformation approaches that can improve HIV care continuum outcomes.

**What did the researchers do and find?:**

- We conducted organizational assessments with clinics at 14 demonstration projects to assess changes in practices and procedures before and after implementation of practice transformations.
- We analyzed Ryan White HIV/AIDS Program Services Report (RSR) data from clinics affiliated with all demonstration projects to examine the impact of practice transformations on client retention in HIV care, prescription of antiretroviral therapy (ART), and viral suppression.
- We found that demonstration project clinics made significant changes in their practices in line with practice transformation approaches to maximize the healthcare workforce involved in the care of people living with HIV (PLWH), promote team-based care, and enhance reliable engagement of clients in primary HIV care.
- We also found that all three practice transformation approaches were associated with improved client retention in HIV care and viral suppression outcomes.

**What do these findings mean?:**

- Practice transformation approaches, particularly those that maximize the HIV care workforce and enhance reliable client engagement in primary HIV care, are of potential benefit for any clinic or healthcare facility that is straining to meet the demands for HIV care with its existing workforce.

## Introduction

The workforce that provides care to people living with HIV (PLWH) (henceforth referred to as the “HIV care workforce”) is at the forefront of the US response to the HIV epidemic. Given the substantial therapeutic and preventive benefits of antiretroviral therapy (ART) [1,2], engaging PLWH in high-quality care is considered key to ending the epidemic [3–5]. The US has made important strides against the HIV epidemic, with new infection rates remaining constant and deaths related to the virus falling in recent years [6]. However, there continue to be ongoing challenges. Nearly 40% of PLWH have not achieved viral suppression, due primarily to failures of linking to and remaining in HIV care [7]. The challenges are made worse by substantial HIV-related disparities seen across demographic characteristics (e.g., race/ethnicity, socioeconomic status) and geographic regions of the country [6,7].

The Ryan White HIV/AIDS Program (RWHAP) is a crucial component of the ongoing US response to the HIV epidemic. Since its inception, the program has ensured that the most vulnerable PLWH have some form of access to care, treatment, and support services [8]. Favorable outcomes among clients in RWHAP-supported settings consistently exceed national statistics, even though the program disproportionately serves the safety net populations least likely to be engaged in HIV care overall [9]. Over 80% of all RWHAP clients are reliably and consistently receiving care, and 86% have achieved viral suppression [9].

Maintaining and improving upon these successes is threatened, however, by projected trends in the healthcare workforce. The US is experiencing a shortage of primary care providers that is expected to increase [10,11]. At the same time, the size of the HIV care workforce is in decline [12] as retirements outpace new entrants into the field. If HIV clinical care delivery continues unchanged, then it will mean that a smaller number of HIV providers and clinical staff—limited by the capacity inherent to current protocols and workflows for care delivery—will face the growing and sustained demand for services, as more PLWH are engaged in care, live longer, and require continuous ART treatment and monitoring. A failure to respond to these dynamics could potentially result in deleterious outcomes, such as reductions in the quality of care (e.g., difficulties securing a timely appointment, less than ideal frequency of medical visits) or burnout and high turnover among the providers and staff who remain.

In 2014, the Health Resources and Services Administration (HRSA) RWHAP Special Projects of National Significance (SPNS) program introduced the System-Level Workforce Capacity Building for Integrating HIV Primary Care in Community Health Care Settings Initiative (henceforth referred to as the Workforce Capacity Building Initiative). Its purpose was to enhance the capacity and readiness of demonstration projects to adapt and realign their workforce and/or practices to improve the provision of quality HIV care. All demonstration projects had existing grants, contracts, or cooperative agreements from the RWHAP to deliver HIV-related medical care and support services. The grants from the SPNS initiative specifically supported efforts to implement practice transformations, which were defined as efficiencies in structures and delivery of care to optimize human resources and improve HIV-related health outcomes. Demonstration projects had broad flexibility in the design of their transformations, potentially tackling workforce challenges by increasing the supply of providers, improving the efficiency of HIV service delivery, and/or facilitating clients’ effective use of care (thereby reducing the need for intensive services to address poorly controlled HIV). In this study, we sought to characterize the practice transformations that were ultimately implemented by the initiative’s demonstration projects and to examine the association of the transformations with retention in HIV care, ART prescription levels, and viral suppression.

## Methods

The details of the methods and findings are presented according to the Strengthening the Reporting of Observational Studies in Epidemiology (STROBE) checklist for cohort studies (S1 Text). The Workforce Capacity Building Initiative issued grants to 15 demonstration projects. Table 1 describes each project and its practice transformation components. Further information about—and lessons learned from—the demonstration project sites can be found in resources prepared by the initiative and posted at HRSA’s TargetHIV website [13–15]. Fourteen of the projects were implemented across an entire agency or at specific clinics within an agency and were intended to shape the way that care is delivered to all HIV clients. A 15th demonstration project, based in Puerto Rico, was focused on ensuring continuity of care by linking clients leaving prisons or jails to community clinics across the island. The unique design of this last project necessitated that it be evaluated differently from the other 14 sites. Therefore, it is not included in the cross-site analyses presented in the rest of this paper.

**Table 1:** Description of practice transformation interventions.

Site Name	Setting	Project Description
ACCESS Community Health Network (Chicago, IL)	Community health centers specialized in HIV (a subset of the larger ACCESS network)	Empanel patients to both an infectious disease specialist and a primary care provider to reduce demands on the specialists. Use care coordinators to support clients’ engagement in care. Implement team-based care and huddles to strategize care planning.
Brightpoint Health (New York, NY)	Network of community health centers	Implement case conferences and provider huddles. Provide self-management courses for patients. Integrate primary care and behavioral healthcare plans through an electronic health record.
Coastal Bend Wellness Foundation (Corpus Christi, TX)	Community health center	Train primary care providers to offer HIV care. Implement multidisciplinary care team meetings and preclinic huddles.
Family Health Centers of San Diego (San Diego, CA)	Multisite network of community health centers, with HIV specialty clinic	Train family medicine providers through a residency program to offer HIV care. Provide care coordination for patients.
Florida Department of Public Health (Osceola County, FL)	Primary care FQHCs located throughout the county and one centralized specialty HIV clinic	Train primary healthcare providers at the county’s FQHCs to be able to manage HIV care patients. Provide ad hoc HIV specialty consultation to primary care providers at FQHCs. Provide opportunity for patients with stable HIV disease to transition care from the HIV specialty clinic to a more conveniently located primary care FQHC.
FoundCare (West Palm Beach, FL)	Community health center with specialty HIV clinic	Provide “warm hand-offs” for patients receiving care in different departments. Implement a care model featuring huddles and team consultation for each patient. Add capacity for psychiatric care and social work.
La Clinica del Pueblo (Washington, DC)	Community health center	Formative assessment to determine highest process and outcome needs in care provision. Continuous quality improvement and iterative internal evaluation cycles to maximize efficient and effective care. Improve cultural competency of clinic staff and providers, particularly with regards to transgender patient population.
MetroHealth (Cleveland, OH)	HIV specialty clinic in academic medical center	Routinely screen and reassess for depression. Treatment and management of depression to be led by care coordinator and consulting psychiatrist.
New York Presbyterian (New York, NY)	HIV clinic in large academic medical center/hospital	Implement panel-based clinical care teams. Facilitate coordination across settings via nurse clinical care coordinators. Implement electronic dashboard to summarize key client outcomes and indicators. Adjust patient flow to be more user friendly and ensure more efficient use of space.
Ruth M. Rothstein CORE Center, CCHHS (Chicago, IL)	Hospital with HIV specialty care clinic; part of a county health and hospitals system	Conduct workflow mapping to address gaps and inefficiencies. Hire CTL to identify and link PLWH to care, help them navigate insurance and the CCHHS health system, as well as health systems outside of CCHHS.
San Ysidro Health (San Diego, CA)	Network of community health centers	HIV 101 trainings across departments in the health center. Patient navigation to assist with referrals. Care team meetings. Residency program to train providers to deliver HIV care.
SHRT (Tyler, Texarkana, and Paris, TX)	Community health centers specialized in HIV	Add family nurse practitioners to HIV clinics so that clinics have the capacity to offer primary care and HIV care. Change helps reduce demands on HIV specialists.
University of Miami (Miami, FL)	HIV clinic in large academic medical center/hospital	Facilitate transitions for patients arriving for appointments or moving from one appointment to another. Link newly diagnosed HIV patients to comprehensive sociomedical support services.
UPMC (site in McKeesport, PA)	Family medicine primary care clinic	Train staff and providers in a family medicine clinic to provide HIV care. Implement a residency training program for family medicine with HIV specialty track.
New York City Health and Hospitals Correctional Health Services (project activities based in Puerto Rico)**	Jails, prisons, community health centers, service agencies	Link PLWH with community-based HIV care and case management directly upon release from incarceration.

**The project based in Puerto Rico was focused at a systems levels, linking clients leaving prisons and jails to community clinics across the island. It did not involve the transformation of practices and personnel within a facility. Because of the project’s unique design, its evaluation had to be structured differently than the methods used at the other demonstration project sites. As such, it is not included in the cross-site analyses presented in this paper.

**Abbreviations:** CCHHS, Cook County Health & Hospitals System; CTL, Clinical Transition Liaison; FQHC, federally qualified health center; PLWH, people living with HIV; SHRT, Special Health Resources for Texas; UPMC, University of Pittsburgh Medical Center.

The University of California San Francisco (UCSF) received a cooperative agreement to conduct a comprehensive evaluation of the demonstration projects’ practice transformations. At the time the application for funding was written, the UCSF team provided a general overview of the procedures we anticipated we would deploy (in the absence of knowing which demonstration projects would be funded or the nature of their practice transformations) and listed the major evaluation goals. Among these was to determine which types of practice transformations would most strongly be associated with changes in key HIV care continuum outcomes, including retention, ART prescription, and viral suppression. In the first year of funding, the exact procedures for the evaluation were further specified. We did not have a prespecified analysis plan.

The finalized cross-site evaluation plan involved multiple components that captured practice transformation implementation and care outcomes and examined changes over time. For the analyses presented here, we focus on 2 specific evaluation components: (1) an organizational assessment that allowed us to characterize structures and practices at the demonstration project sites and (2) Ryan White HIV/AIDS Program Services Report (RSR) data capturing key HIV care engagement and clinical outcomes. Procedures for the cross-site evaluation were reviewed and approved by the UCSF Institutional Review Board (IRB; Protocol #15–16326). The organizational assessment was used to gather data only about organizations, not individuals, and was thus deemed by the UCSF IRB not to be research with human participants. As such, a formal consent process was not required. Nonetheless, we opted to obtain verbal consent from the project lead at each demonstration site to collect data about their organization. The RSR data were supplied as a limited dataset as defined under the Health Insurance Portability and Accountability Act (HIPAA). The files contained exact dates and locations of clinical services but did not contain other personal health identifiers. In alignment with the governing provisions of HIPAA, the UCSF IRB allows a limited dataset to be used for research purposes without obtaining verbal or written consent from the clients who contributed information to the dataset.

### Organizational assessment

The organizational assessments were conducted with each of the 14 demonstration sites at baseline—immediately prior to the launch of the site’s practice transformation—and then twice annually for 2 years following implementation, for a total of 5 assessments. Team leads from each of the demonstration project sites completed the assessments in consultation with other team members as needed. All responses were entered into a Research Electronic Data Capture (REDCap) database hosted at the university [16]. Demonstration project team leads also independently completed a short REDCap survey for each assessment wave through which they reported basic information about their clinical sites, including the number of patients served and number of prescribing providers.

The organizational assessment consisted primarily of the Building Blocks of Primary Care Assessment (BBPCA), which was developed by the UCSF Center for Excellence in Primary Care [17]. It focuses on 10 aspects of patient-centered care. Because the BBPCA covers general primary care, we created supplemental items to assess practices specific to HIV care. Where possible, the wording of these items mirrored language used in comparable items of the original BBPCA. For the analyses presented here, we focus on 3 of the HIV-specific “blocks.” These blocks were developed to capture changes consistent with major approaches for addressing workforce shortages. At the outset of the initiative (prior to implementation or evaluation activities), the UCSF team reviewed demonstration project plans and determined that the proposed practice transformations fell into 3 approaches that were not mutually exclusive. First, there was a set of transformations intended to maximize the HIV care workforce. Because demonstration projects received RWHAP money to deliver care, they already had some providers and staff who served PLWH. Transformations to maximize the HIV care workforce thus focused on increasing the number of existing providers and staff who could contribute to the care of PLWH. This generally took 2 forms: (a) the expansion of HIV services to a larger number of clinical facilities within a regional care network that previously had most HIV services housed in a limited number of clinics or (b) expansion of the number of providers serving PLWH in an individual facility. In both cases, sites began their projects with an existing care model in which HIV specialists were responsible for delivering almost all medical care to PLWH. The local practice transformations thus included efforts to increase the involvement of general primary care providers and other relevant providers in the services that PLWH received. A second set of transformations promoted share-the-care, which involved enhancing capacities and responsibilities of midlevel providers and clinical staff to handle more routine aspects of care, thereby freeing up primary HIV care providers to deal with more complex situations. These transformations frequently involved the initiation or augmentation of team-based care models. Finally, a third group of transformations were designed to facilitate more efficient, effective, and reliable use of HIV primary care by clients through services like care coordination. This last approach was intended to address workforce challenges indirectly. By increasing clients’ use of primary HIV care, the need for resource- and time-intensive urgent, emergency, and/or inpatient care should ideally be reduced.

Each of the HIV-specific blocks of the organizational assessment captured a specific transformation approach. The first block consisted of 2 items related to maximizing the HIV care workforce: (1) whether a practice is able to offer advanced HIV care and (2) whether HIV services are integrated into general primary care clinics. The second block contained 3 items assessing share-the-care, capturing information about (1) the utilization of team-based care, including workflows for clinical teams, (2) routine assessment of training needs for both providers and staff, and (3) use of standing orders. The third block contained 4 items focused on efforts to reliably engage clients in care, capturing procedures related to (1) population management and coordination of care practices, including tracking and intervention with PLWH who are referred to the site but who do not enroll, (2) tracking and follow-up with enrolled clients who are overdue for care, (3) linking PLWH to supportive wraparound services, and (4) clinical care management services for high-risk PLWH. It should be noted that the items within each block were grouped conceptually based on their intent and captured strategies that would mutually support and reinforce a particular practice transformation if deployed together. A clinic could strive toward the goal of a transformation strategy, however, by deploying changes reflected in only a subset of the relevant block’s items.

**Fig 1:**
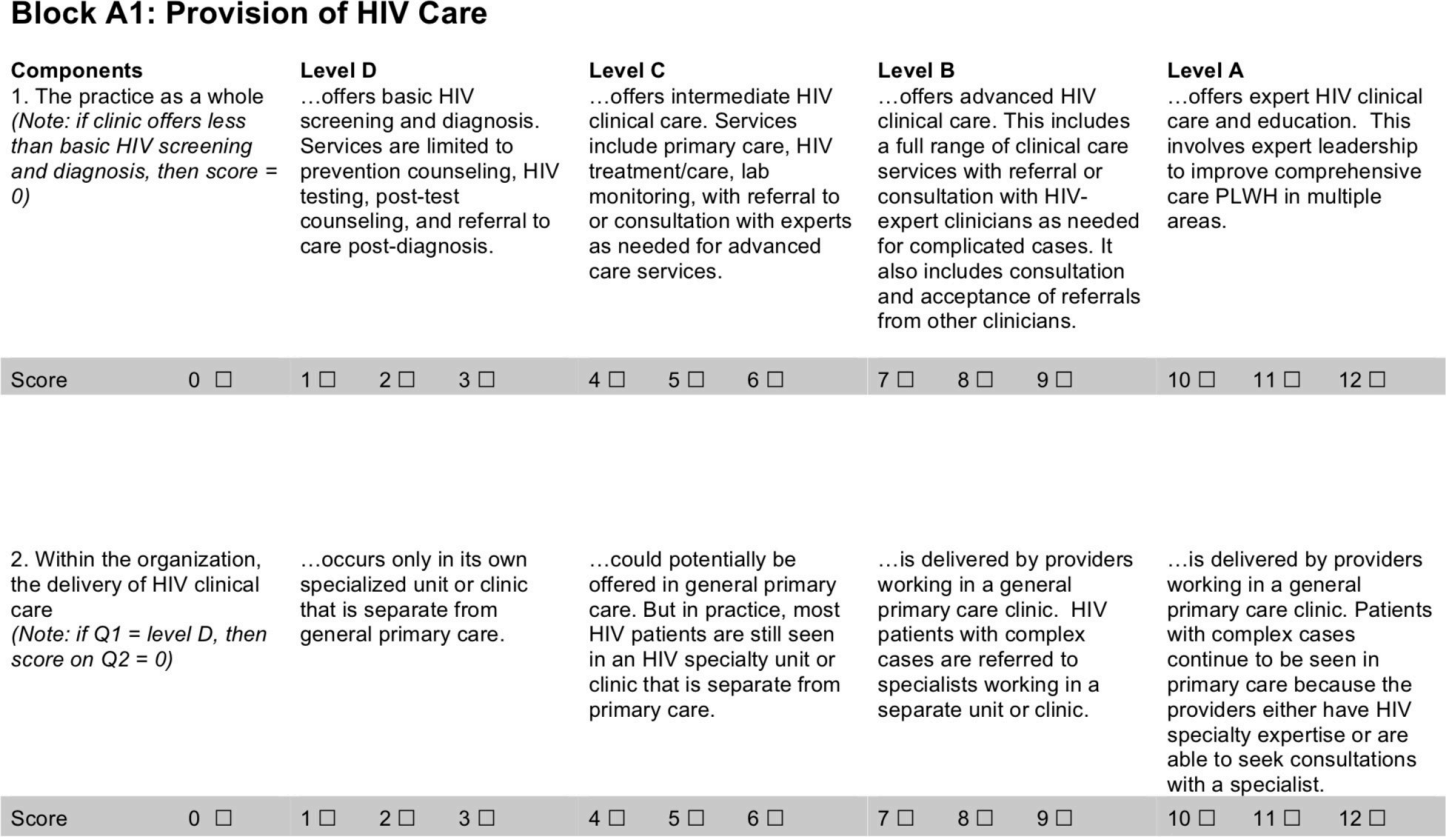
Example of HIV-specific supplemental items from the organizational assessment. PLWH, people living with HIV.

For each item, the demonstration projects teams were presented with 4 descriptions that characterized potential practices pertaining to a facet of care delivery. See Fig 1 for an example of the layout for 2 of the items. The complete set of organizational assessment items used in the analyses can be found in Supporting Information (S2 Text). The descriptions in each item always ranged from one indicative of low capacity or limited integration of HIV care to one indicative of high capacity or strong integration of HIV care into general practice. The other two descriptions captured intermediary practices between the two extremes. Site personnel selected a numerical score aligned with the description that best characterized the current practices at their site. Numerical responses could range from 1 to 12, with three numbers grouped under each of the four descriptions. The four descriptions provided a guide for the respondents when selecting an answer. Our analyses worked only with the continuous 12-point response scale. Higher numbers reflected greater capacity or stronger integration of HIV care into primary care. This setup, which was part of the original BBPCA’s design, allowed for tracking not only major changes in practices but also more minor ones (e.g., gradual expansion of practice transformation changes to more clinics within a facility).

To ensure comparability in the use of the response scale and to guard against inflation of the scores, all organizational data were collected by UCSF investigators during scheduled data collection calls with each demonstration project team. At the time of an assessment, a project team gave its initial score for each item and then was asked to explain the logic behind the score. UCSF investigators were able to provide common guidance on how to decide among different score options for the items. Furthermore, UCSF separately conducted site visits and collected additional forms of data (not featured in this report), including detailed qualitative interviews with project personnel, to understand and characterize the kinds of changes made at each site. These independent forms of observation reduced the incentive for a site to misrepresent or otherwise overstate its project’s progress on the organizational assessment.

### RSR data

Client-level outcomes were assessed using data from the RSR for each site. The RSR consists of demographic, HIV care, and treatment information. Each year, all recipients and subrecipients of RWHAP grants, contracts, and cooperative agreements are required to submit an RSR to HRSA. Prior to 2015, the RSR captured information only on people who had received services that were paid for by the RWHAP. Starting in 2015, it captured information on all of an agency’s clients who are living with HIV, including those whose care and services were supported by other entities, such as Medicaid, Medicare, or private insurance. UCSF established a data-sharing agreement with HRSA to obtain copies of the RSR data from the demonstration sites. Three calendar years (CYs) of RSR data were included: CY2014 reflected clinical services and outcomes before practice transformations were implemented, CY2015 captured outcomes as transformations were ongoing, and CY2016 captured outcomes in the year following transformations. All data were securely transferred using a university-hosted, HIPAA-compliant data portal. The files contained the standard de-identified encrypted unique client identifier (eUCI) that is created for each RSR record following rules established by HRSA.

The RSR data files contained information on all clients reported by an organization for each year. These records potentially included a broader set of individuals than those affected by a project’s practice transformations (e.g., some RSR records were submitted by an agency on behalf of its full consortium of clinics, but the local demonstration project was being implemented in only a couple of the clinics). To ensure that the right set of client records were used for analyses, UCSF obtained lists of eUCIs directly from each demonstration site for clients who were receiving care in facilities where practice transformation activities were occurring. These eUCIs allowed us to subset the relevant clients from the RSR data files sent by HRSA.

### Analyses

Individual items within each block of the organizational assessment were averaged to create a total score for the block. If a demonstration project had multiple clinics with differing scores for an item, the average response across clinics was calculated before generating the total block score. Generalized estimating equation (GEE) models, clustered by site with repeated measures, were used to analyze the change over time from baseline to the final assessment. Prior to estimating changes over time, the scores were rescaled from 1–12 to 0–100 so that all change estimates reflected the total percent change in scores. For example, a 25% change estimate would indicate a 3-point increase on the original 12-point scale. We separately examined to what degree individual items in each block correlated with the overall scores for the block. This step allowed us to determine whether specific practice changes were particularly influential in changes observed in the block scores.

We used responses from the organizational assessment to determine which practice transformation approaches were effectively implemented by each demonstration site. For each block, a site was categorized as having or not having transformed relevant clinical workflows and practices based on whether scores for the block changed significantly over time. The categorizations were not mutually exclusive, as practices within a site could potentially be transformed in more than one block. We based the categorizations on observed changes, as opposed to assigning sites to categories based on their plans at project start, because the sites were permitted to use iterative, quality improvement approaches to enact practice changes. As a result, the specifics of the transformations at each site potentially evolved over the course of the initiative.

For the RSR data, we assessed changes over time for 3 key outcomes: retention in care (proportion of clients with at least one medical visit in each 6-month period of the CY, with at least 60 days between visits), prescription of ART (proportion of clients prescribed ART at any point during the year), and viral suppression (proportion of clients with <200 copies/mL at last test). These outcomes correspond to major milestones of the HIV care continuum [4]. We did not utilize 2 other common milestones of the continuum—diagnosis and initial linkage to care—as the practice transformations at the sites were principally intended to shape the care of those who were already engaged in care. Our definitions for the outcomes were aligned with HRSA performance measures [18], with the exception that the retention measure was based on data from a 12-month period rather than a 24-month period to facilitate the examination of changes across CYs. GEE with repeated measures clustered by client were used to model differences and estimate proportions of each outcome over time. These multivariate models included inverse probability weights to adjust for demographic differences between and within sites from year to year. Demographic variables used to create the inverse probability weights included age, race, insurance type, and HIV risk factors. A population weight was also included to adjust for eligible clinic population size relative to the number of eligible patients reported by the demonstration site. The models allowed us to assess changes over time for the 3 outcomes across all sites, as well as for the set of sites that implemented each specific practice transformation approach. All data management and analyses were conducted using SAS version 9.4 (SAS Institute, Cary, NC). An alpha level of 0.05 was used for all comparisons.

## Results

The 14 demonstration project sites reported a mean of 3,089 clients (SD = 2,932; median 2,333; interquartile range [IQR] 1,729–4,021) in the 6-month period preceding their baseline organizational assessment. Of these, a mean of 916 (SD = 1,167; median 465; IQR 237–1,400) were PLWH. The project sites had a mean of 18.5 prescribing providers (SD = 23.5; median 9; IQR 5–25). At most sites, at least some of the providers were part-time employees. As a result, the total amount of provider personnel time, when expressed as an equivalent number of full-time positions, was lower (mean 7.7; SD = 7.5, median 5.25; IQR 3–13.2). The only near significant change over time in demonstration project characteristics was the average number of clinics per site (baseline assessment wave: mean 2.1; SD = 1.5; median 1; IQR 1–3; final assessment wave: mean 1.9; SD = 1.5; median 1; IQR 1–3; *p* = 0.051).

There were 13,571 clients reflected in the RSRs across sites in CY2014 (mean 969/site; SD = 1,351; median 459.5; IQR 134–1,186). This number rose to 15,083 for CY2015 (mean 1,077; SD = 1,265; median 742; IQR 257–1,009) and 15,738 for CY2016 (mean 1,124; SD = 1,243; median 844; IQR 301–1,018). In CY2014, clients were 71% male, 28% female, and 0.2% transgender. A plurality (48%) were African American. Almost one-third (31%) were Hispanic/Latino, and 14% were white. The mean age was 47 (IQR 37–54). Equal proportions were heterosexual men or women, or men who have sex with men (39% each), while 7% were injection drug users. Forty-one percent were on Medicaid, 11% were on Medicare, and 29% had no insurance. There were no significant differences over time in either the population size or the client demographics, even though the reporting instructions for the RSR changed from CY2014 to CY2015 (shifting from including only clients receiving RWHAP-supported services to including all clients living with HIV). The lack of difference is likely due to the fact that most patients living with HIV were already included in the RSR reports prior to the change in reporting instructions (e.g., because they were receiving RWHAP-funded, nonmedical support services).

**Fig 2:**
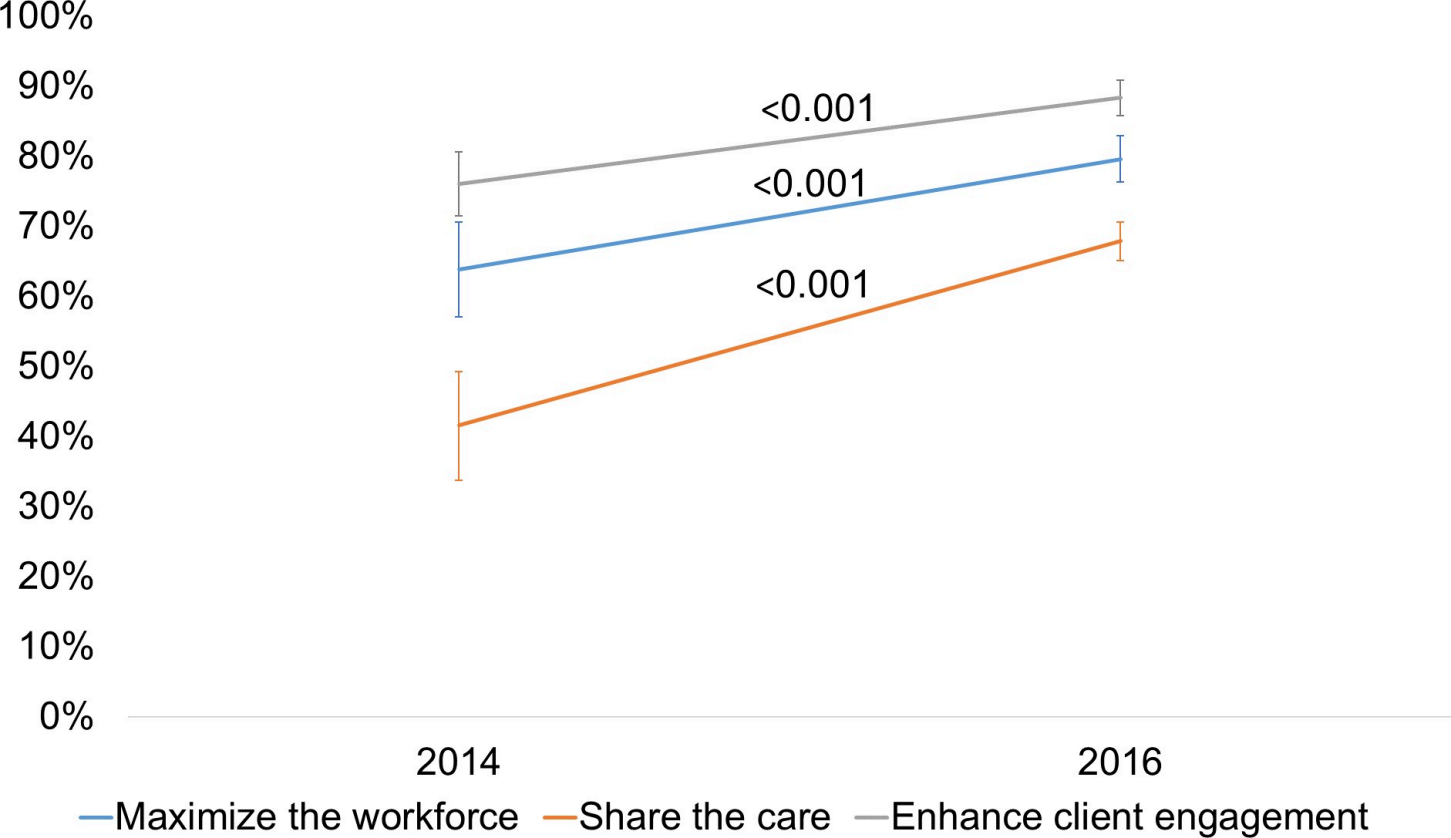
Changes in organizational assessment block scores from baseline to final assessment wave.

Fig 2 displays the changes over time in block scores for the organizational assessments. There were significant increases in capacities and practices consistent with maximizing the HIV care workforce (18% change, *p* < 0.001), share-the-care (25% change, *p* < 0.001), and facilitating clients’ reliable engagement in HIV primary care (13% change, *p* < 0.001). Overall, 6 of the sites reported significant changes intended to maximize the HIV care workforce, 9 had significant changes in share-the-care practices, and 4 significantly changed practices to facilitate client engagement in primary HIV care. Across the 14 sites, 36% (*n* = 5) had changes in scores in 2 or more of the blocks while 43% (*n* = 6) had changes in 1 block only. There were 3 sites that had no significant change in any of the 3 organizational assessment blocks.

Inspection of the individual items that composed each block suggested the there were differences in whether practice changes consistent with a transformation strategy were implemented together or individually. Transformations to enhance client engagement showed the most robust evidence of being implemented consistently as a package. The overall scores for this block were strongly correlated with the block’s individual items assessing systematic tracking and follow-up of patients newly referred to a practice for HIV care (*r* = 0.761), linking HIV clients to wraparound services (*r* = 0.840), and systematic provision of clinical management services for high-risk clients (*r* = 0.774). The fourth item in the block measuring tracking and intervening with clients overdue for HIV care showed a smaller, albeit still strong, association with the overall score (*r* = 0.547). By contrast, the findings for share-the-care transformations suggested that the block’s scoring was driven heavily by one particular kind of practice change. Specifically, the item assessing whether a clinic was routinely monitoring and responding to provider and staff training needs was correlated more strongly with the overall block score (*r* = 0.539) than the items assessing deployment of HIV clinical workflows (*r* = 0.126) or use of standing orders (*r* = 0.267). Finally, the data pertaining to transformations to maximize the workforce suggested the use of several successful approaches. The 2 items for this block correlated only moderately with one another (*r* = 0.193), with sites differing in the degree of focus they placed upon enhancing HIV expertise among providers versus integration of HIV care and primary care.

**Fig 3:**
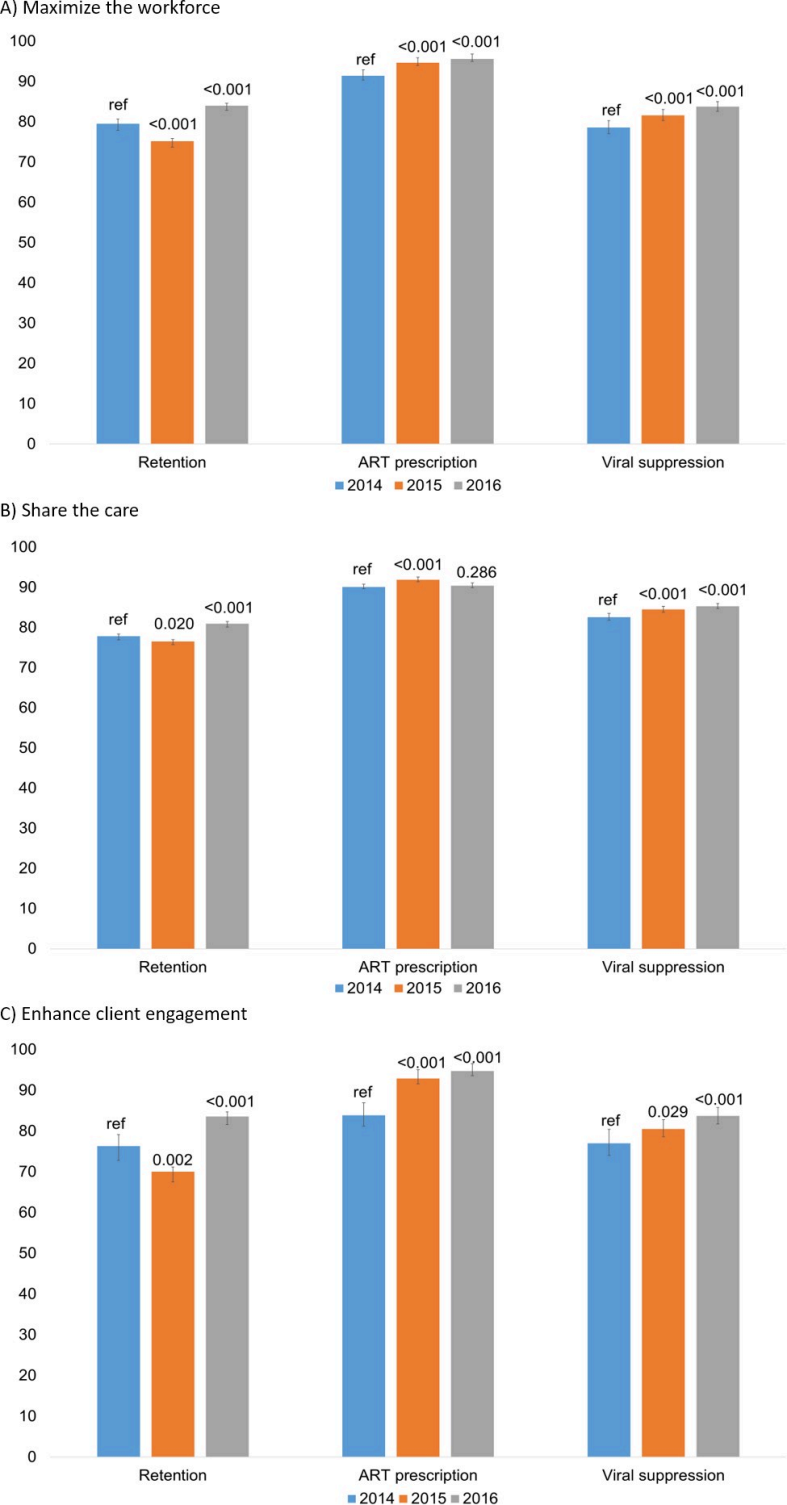
Changes in care continuum outcomes over time for demonstration project sites implementing each practice transformation approach. ART, antiretroviral therapy.

Across sites, there were significant improvements from CY2014 to CY2016 in the 3 major outcomes assessed using RSR data. Retention in HIV care increased from 78.5% to 81.4% (adjusted odds ratio [aOR] = 1.03; 95% confidence interval [CI] 1.02–1.04; *p* < 0.001), ART prescription levels increased from 90.6% to 91.4% (aOR = 1.01; 95% CI 1.00–1.01; *p* = 0.006), and viral suppression increased from 80.1% to 83.1% (aOR = 1.03; 95% CI 1.02–1.04; *p* < 0.001). Fig 3 displays the outcomes for the subsets of sites that implemented each type of practice transformation. Among those sites that maximized the HIV care workforce, there were significant improvements in retention in care (CY2014: 79.5%; CY2016: 84.0%; aOR = 1.05; 95% CI 1.02–1.07; *p* < 0.001), ART prescription levels (CY2014 91.4%; CY2016: 95.6%; aOR = 1.04; 95% CI 1.03–1.05; *p* < 0.001), and viral suppression (CY2014: 78.6%; CY2016: 83.8%; aOR = 1.05; 95% CI 1.04–1.07; *p* < 0.001). Similarly, at project sites that changed client engagement practices, there were significant improvements in retention in care (CY2014: 76.3%; CY2016: 83.6%; aOR = 1.08; 95% CI 1.03–1.12; *p* < 0.001), ART prescription levels (CY2014: 83.9%; CY2016: 94.7%; aOR = 1.11; 95% CI 1.08–1.14; *p* < 0.001), and viral suppression (CY2014: 77.0%; CY2016: 83.7%; aOR = 1.07; 95% CI 1.03–1.10; *p* < 0.001). By contrast, at the demonstration projects that altered share-the-care practices, there were smaller but still significant improvements in retention in care (CY2014: 77.8%; CY2016: 80.9%; aOR = 1.03; 95% CI 1.02–1.04; *p* < 0.001) and viral suppression (CY2014: 82.6%; CY2016: 85.3%; aOR = 1.03; 95% CI 1.02–1.04; *p* < 0.001). There were no statistically significant changes in ART prescription levels at these sites (CY2014: 90.1%; CY2016: 90.4%; aOR = 1.00; 95% CI 0.99–1.01; *p* = 0.280). At the small number of demonstration project sites that did not successfully implement any of the 3 practice transformation strategies, there were statistically significant improvements in viral suppression only (CY2014: 75.2%; CY2016: 78.3%; aOR = 1.03; 95% CI 1.01–1.05; *p* = 0.002). There were no changes in retention or ART prescription levels (*p* = 0.142 and *p* = 0.381, respectively).

## Discussion

Our cross-site evaluation of the SPNS Workforce Capacity Building Initiative showed that RHWAP-supported demonstration project sites were able to implement practice transformations that addressed workforce challenges using 3 major approaches: (1) maximizing the existing healthcare workforce involved in the care of PLWH, (2) adopting share-the-care practices to optimize the use of midlevel providers and clinical staff, and (3) implementing client engagement strategies to improve the reliable use of primary HIV care services. Approaches to maximize the HIV care workforce and enhance client engagement in care were associated with significant improvements over time in 3 major HIV care continuum outcomes: retention in HIV care, ART prescription levels, and viral suppression. By contrast, share-the-care transformations were associated with smaller improvements in retention and viral suppression, and with no changes in ART prescription levels.

Share-the-care transformations were adopted by the largest number of sites. The largest increase in the overall block score measuring practice transformation-related changes was also seen with this approach. By contrast, client engagement transformations were implemented at the fewest sites and involved the smallest change in the overall block score measuring practice transformation. These observed differences in frequency and magnitude of changes are likely due in part to the nature of the different transformations themselves. For sites that face substantial shortfalls in their workforce capacity, maximizing the HIV care workforce and share-the-care transformations are arguably more logical choices. These approaches tackle workforce challenges directly by expanding capacity through increased supply or efficiency of care. They are also intended to have wide impact, with all clients potentially experiencing direct or indirect improvements in care as a result of the changes. By contrast, programs to enhance client engagement tackle workforce challenges more indirectly. They seek to enhance clients’ use of primary HIV care in order to avert the need for resource-intensive urgent or emergency care as a result of poorly controlled HIV. The changes in this type of transformation are also more targeted, with new services being directed primarily at PLWH who are at higher risk of falling out of care or who have not successfully engaged in care. Furthermore, in order for client engagement approaches to be effective, a site must first ensure that there is adequate supply of care to which a client can be directed.

Inspection of the individual items used to measure each type of practice transformation provide important information about the kinds of changes that demonstration projects used to achieve their successes. Two items, one measuring HIV expertise among providers and one measuring integration of HIV and primary care, contributed to the assessment of transformations to maximize the HIV workforce. Their scores were correlated only modestly. This is likely a reflection of the different strategies pursued by the demonstration projects that successfully implemented relevant transformations. Some focused on raising capacity to deliver HIV care at new clinics within a regional health system, a goal more aligned with improving HIV-related skill and expertise, whereas others already had HIV specialty practices and sought to expand patients’ utilization of other types of providers (e.g., use of primary care providers for more routine HIV care needs), a goal more heavily focused on integration of HIV and primary care services. Our data suggest that both strategies can successfully contribute to expanding the proportion of the overall care workforce able to serve patients living with HIV. By contrast, we found that observed changes in share-the-care transformations were driven heavily by the deployment of strategies to monitor and respond to training needs among a clinic’s providers and staff. This finding highlights the critical role that training plays in practice changes that involve task shifting. Such approaches fundamentally seek to increase the responsibilities of clinical staff and midlevel providers, and it is vital that they be equipped fully with the capacity to succeed in their enhanced role. Share-the-care transformations were less heavily driven by deployment of HIV care workflows and use of standing orders. The lower association with HIV care workflows may simply reflect relatively lower usage of this strategy among our demonstration projects. The lower association with standing orders is possibly due to multiple demonstration projects being located in jurisdictions where the use of some or all standing orders was precluded by law. Finally, our findings for transformations to enhance client engagement suggest that implementation success involves a focus on multiple strategies. Tracking newly referred patients, providing clinical care management for high-risk patients, and linking patients to supportive services all strongly contributed to changes in measured scores. These findings speak to the importance of providing comprehensive support for patients who struggle to remain in care.

The observed associations among practice transformation approaches and HIV care outcomes should be considered in the context of national trends. During the same time period (CY2014 to CY2016), there were increases in retention in care (80.4% to 81.7%) and viral suppression (81.4% to 84.9%) among all clients in RWHAP settings across the country [9]. In our study, the observed changes in these 2 outcomes exceeded the national trends at the demonstration projects that implemented transformations to maximize the HIV care workforce or enhance client engagement, with an increase of nearly 5 percentage points or more for each outcome at clinics implementing each transformation strategy. By contrast, at sites implementing share-the-care transformations, the change in retention in care exceeded the national trend, while the change in viral suppression did not (approximately 3-percentage-point increase in each outcome). It is also worth noting that, among sites that did not successfully implement any of the major practice transformation approaches, the change in viral suppression was of similar magnitude to the change reported nationally [9]. These observations suggest, but do not allow us to definitively conclude, that transformations to maximize the HIV care workforce and enhance client engagement substantively improved multiple HIV care outcomes over and above any contemporaneous temporal trends, while share-the-care approaches were more limited to improvements in retention in care.

We recognize that the overall magnitude of the observed improvements remains small. But it is important to note that outcomes at our demonstration projects, like outcomes in the RWHAP more generally, were strong at the project outset. Statistically, this creates a ceiling effect that makes increasingly desirable outcomes harder to achieve (i.e., one is working against regression to the mean). In the context of this study, such a pattern is best understood by considering who benefits most from a practice transformation. The patients who require the least intervention to stay engaged in care were likely the ones already successfully engaged when the project began. Thus, the demonstration projects were striving to improve outcomes among those patients who are relatively harder to engage. This group inevitably requires more effort just to achieve smaller improvements because the highest-risk patients may face numerous structural, interpersonal, and individual barriers to successful engagement in care. Although large changes in outcomes with this group can be harder to demonstrate, a failure to meet their needs would result in them being left behind in efforts to control and end the HIV epidemic.

Within the context of our study, the generally weaker changes in care continuum outcomes for share-the-care transformations may be due to the nature of the transformations themselves. Approaches to maximize the HIV care workforce or implement client engagement strategies are potentially targeting known gaps in the services being delivered. By contrast, share-the-care transformations shift responsibility for tasks. These transformations set the stage for a clinic to deliver care more efficiently, which potentially allows for more clients to be served. But at the time a share-the-care transformation is implemented, it is possible that there is no change in whether a service is delivered to each client, just in how and by whom it is delivered. Importantly, the unchanged ART prescription level outcomes and the small improvements in retention and viral suppression at sites implementing share-the-care transformations suggest that shifting responsibilities away from primary HIV care providers and toward midlevel providers and clinical staff did not compromise the quality of HIV care.

The absence of a statistically significant change in ART prescription levels at sites implementing share-the-care transformations is not necessarily surprising given high ART prescription levels among these sites at the start of the project. The finding may also be due to the way that the ART prescription outcome is defined. The indicator reflects the presence of any ART prescription for each client over the course of a year [18] rather than capturing ongoing and reliable use of ART throughout the year. Future studies of practice transformation that use ART adherence as an outcome may show changes more in line with those observed for the retention and viral suppression outcomes in the current study.

Temporal trend data (reflected in Fig 3) consistently showed a small dip in retention in CY2015, which corresponds to right after practice transformations were initiated. In 2016, the outcome then rebounded and substantially improved upon 2014 performance. This U-shaped pattern may be the byproduct of the complex, iterative implementation processes deployed at sites. Given the complexity of some of the practice changes, demonstration projects did not necessarily launch all transformation components at once. Even once launched, some aspects took time to reach their full potential (e.g., monitoring and addressing training needs). And, at some sites, there were tweaks to protocols and workflows in response to unanticipated challenges. Collectively, these iterations could have led to temporary decreases in performance until the transformed practices were fully implemented, after which an increase in performance was seen.

Our results have important implications for RWHAP-supported and other healthcare settings in the US. The country has placed concerted effort on reducing HIV-related disparities and ensuring that the vast majority of PLWH are in care and are virally suppressed. This can be seen in efforts to meet the goals of the National HIV/AIDS Strategy [5] and in the release of the Department of Health and Human Services’ new “Ending the HIV Epidemic Initiative: A Plan for America” [19]. The latter intends to bring down new infections by first focusing on the highest-impacted regions of the country and subsequently expanding efforts to the nation as a whole [19]. Achieving the goals of the Ending the HIV Epidemic Initiative will not be possible (or will be short-lived) if there are increasing shortfalls in the availability of high-quality HIV care. Importantly, the practice transformation approaches implemented in this SPNS project build on strategies that are being used in other contexts. For example, training components of the maximizing the HIV care workforce transformations align with longstanding efforts to ensure quality care by existing HIV providers [20,21]. The trainings implemented in this initiative were novel primarily because of their audience (providers and staff at the demonstration project sites who historically were not involved in delivering care to PLWH) and because of where the programs were implemented (e.g., new tracks in family medicine residencies). Share-the-care transformations are consistent with efforts to implement task shifting or task sharing [22,23]. Client engagement strategies are aligned in spirit and practice with increasingly popular interventions like patient navigation [24,25]. What was unique and of value in this initiative was demonstration project sites’ flexibility to mix and match practice transformation approaches to achieve significant improvements in outcomes. Widespread and flexible use of such strategies could potentially enable high-quality HIV care to be delivered to more PLWH. Practice transformations may also help to blunt any deleterious effects resulting from declining numbers of HIV and primary care providers [10,12].

Our results are tempered by several limitations. First, all demonstration projects were being supported by the RWHAP to deliver HIV care and support services (in addition to receiving grants for this specific project). RWHAP clinics are known to achieve better-than-average HIV care continuum outcomes [9] and to offer more comprehensive support services than other settings [26]. As such, it is possible that some of our observed outcomes may not generalize to non-RWHAP settings. Second, the use of a demonstration project design for the initiative limited the amount of control we had over which practice transformations were implemented. Most sites implemented more than one practice transformation approach. They also varied considerably in the exact procedures used to achieve the different approaches. Although we were able to examine outcomes at sites that implemented each transformation approach, the comparisons are less rigorous than they would be in a randomized controlled trial (RCT) in which each site is assigned to use only one approach and to follow tightly specified protocols for achieving the desired changes. Third, although we are able to compare our findings against national data [9], we cannot draw rigorous conclusions about the role of contemporaneous temporal trends in our findings because we did not have true control condition as a point of comparison. Finally, there is the possibility of a Hawthorne effect, whereby clinical personnel who knew that they were under study were more motivated to make their projects a success.

Practice transformation is a potentially useful strategy for addressing anticipated workforce challenges among those providing care to PLWH. It holds the promise of optimizing the use of personnel and ensuring the delivery of care to all who need it, while not compromising—and potentially even enhancing—HIV care continuum outcomes. Approaches that maximize the HIV care workforce and enhance reliable client engagement in primary HIV care are of potential benefit for a clinic or healthcare facility that is straining to meet the demands for HIV care with its existing workforce. Approaches that promote share-the-care may have more limited impact on HIV care outcomes, specifically by improving retention in care. Future research should examine the use of practice transformations to address other areas of healthcare delivery affected by workforce shortages and to further optimize the potential benefits of such transformations for the delivery of HIV care.

## Supporting information

S1 Text STROBE statement—checklist of items that should be included in reports of cohort studies. STROBE, Strengthening the Reporting of Observational Studies in Epidemiology.
(DOC)

S2 Text Organizational assessment addendum items used in the analyses. (DOCX)

## Abbreviations

aOR: adjusted odds ratio
ART: antiretroviral therapy
BBPCA: Building Blocks of Primary Care Assessment
CCHHS: Cook County Health & Hospitals System
CI: confidence interval
CTL: Clinical Transition Liaison
CY: calendar year
eUCI: encrypted unique client identifier
FQHC: federally qualified health center
GEE: generalized estimating equation
HIPAA: Health Insurance Portability and Accountability Act
HRSA: Health Resources and Services Administration
IQR: interquartile range
IRB: Institutional Review Board
PLWH: people living with HIV
REDCap: Research Electronic Data Capture
RCT: randomized controlled trial
RSR: Ryan White HIV/AIDS Program Services Report
RWHAP: Ryan White HIV/AIDS Program
SHRT: Special Health Resources for Texas
SPNS: Special Projects of National Significance
STROBE: Strengthening the Reporting of Observational Studies in Epidemiology
UCSF: University of California San Francisco
UPMC: University of Pittsburgh Medical Center
