# Supplementary material for: Practice transformations to optimize the delivery of HIV primary care in community healthcare settings in the United States: A program implementation study
Source: PLoS Med. 2020 Mar 26;17(3):e1003079. doi: 10.1371/journal.pmed.1003079 (PMC7098549; doi:10.1371/journal.pmed.1003079)
Supplement: S2 Text — (DOCX) [file pmed.1003079.s002.docx]

**S2 Text. Organizational Assessment Items Used in the Analyses**

**MAXIMIZING THE HIV WORKFORCE BLOCK**

| **Components** | **Level D** | **Level C** | **Level B** | **Level A** |
| --- | --- | --- | --- | --- |
| 1. The practice as a whole *(Note: if clinic offers less than basic HIV screening and diagnosis, then score = 0)* | …offers basic HIV screening and diagnosis. Services are limited to prevention counseling, HIV testing, post-test counseling, and referral to care post-diagnosis. | …offers intermediate HIV clinical care. Services include primary care, HIV treatment/care, lab monitoring, with referral to or consultation with experts as needed for advanced care services. | …offers advanced HIV clinical care. This includes a full range of clinical care services with referral or consultation with HIV-expert clinicians as needed for complicated cases. It also includes consultation and acceptance of referrals from other clinicians. | …offers expert HIV clinical care and education. This involves expert leadership to improve comprehensive care PLWH in multiple areas. |
| Score 0 ☐ | 1 ☐ 2 ☐ 3 ☐ | 4 ☐ 5 ☐ 6 ☐ | 7 ☐ 8 ☐ 9 ☐ | 10 ☐ 11 ☐ 12 ☐ |
|  |  |  |  |  |
| 2. Within the organization, the delivery of HIV clinical care  *(Note: if Q1 = level D, then score on Q2 = 0)* | …occurs only in its own specialized unit or clinic that is separate from general primary care. | …could potentially be offered in general primary care. But in practice, most HIV patients are still seen in an HIV specialty unit or clinic that is separate from primary care. | …is delivered by providers working in a general primary care clinic. HIV patients with complex cases are referred to specialists working in a separate unit or clinic. | …is delivered by providers working in a general primary care clinic. Patients with complex cases continue to be seen in primary care because the providers either have HIV specialty expertise or are able to seek consultations with a specialist. |
| Score 0 ☐ | 1 ☐ 2 ☐ 3 ☐ | 4 ☐ 5 ☐ 6 ☐ | 7 ☐ 8 ☐ 9 ☐ | 10 ☐ 11 ☐ 12 ☐ |
|  |  |  |  |  |

| **SHARE-THE-CARE BLOCK** | | | | |
| --- | --- | --- | --- | --- |
|  |  |  |  |  |
| **Components** | **Level D** | **Level C** | **Level B** | **Level A** |

| 1. HIV care workflows for clinical teams | ...have not been documented and/or are different for each person or team. | … have been documented, but are not used to standardize workflows across the practice. | ... have been documented and are utilized to standardize practice. | ...have been documented, are utilized to standardize workflows, and are evaluated and modified on a regular basis. |
| --- | --- | --- | --- | --- |
| Score | 1 ☐ 2 ☐ 3 ☐ | 4 ☐ 5 ☐ 6 ☐ | 7 ☐ 8 ☐ 9 ☐ | 10 ☐ 11 ☐ 12 ☐ |

| 2. The practice | ...does not have an organized approach to identify or meet the HIV-related training needs for providers and other staff | … routinely assesses HIV-related training needs and assures that staff are appropriately trained for their roles and responsibilities in HIV care. | ... routinely assesses HIV-related training needs, assures that staffed are appropriated trained for their roles and responsibilities in HIV care, and provides some cross-training to permit staffing flexibility. | ... routinely assesses HIV-related training needs, assures that staffed are appropriated trained for their roles and responsibilities in HIV care, and provides cross-training to assure that patient needs are consistently met. |  |  |  |  |  |
| --- | --- | --- | --- | --- | --- | --- | --- | --- | --- |
| Score | 1 ☐ 2 ☐ 3 ☐ | 4 ☐ 5 ☐ 6 ☐ | 7 ☐ 8 ☐ 9 ☐ | 10 ☐ 11 ☐ 12 ☐ |  |  |  |  |  |
| 3. Standing orders for HIV-related care that can be acted on by non-physicians under protocol | ...do not exist for the practice. | … have been developed but are not regularly used. | ... have been developed and are regularly used. | ... have been developed and are used extensively. |  |  |  |  |  |
| Score | 1 ☐ 2 ☐ 3 ☐ | 4 ☐ 5 ☐ 6 ☐ | 7 ☐ 8 ☐ 9 ☐ | 10 ☐ 11 ☐ 12 ☐ |  |  |  |  |  |

| **ENHANCING CLIENT ENGAGEMENT BLOCK** | | | | |
| --- | --- | --- | --- | --- |
| **Components** | **Level D** | **Level C** | **Level B** | **Level A** |
| 1. HIV-positive individuals who are referred to the practice but do not enroll in care | ...would not be identified. | …could potentially be identified and tracked under certain circumstances (e.g., have agreement/ relationships with referring agencies). But identification happens inconsistently. | ...are systematically identified whenever possible (e.g., have agreement/relationships with referring agencies and defined processes to identify referrals and missed visits). Follow-up with such individuals is inconsistent and/or limited (e.g., telephone reminder). | ...are systematically identified whenever possible (e.g., have agreement/relationships with referring agencies and defined processes to identify referrals and missed visits) AND a staff member is consistently assigned the task of following-up with such individuals and available to provide a “warm hand-off” to help link them to care. |
| Score | 1 ☐ 2 ☐ 3 ☐ | 4 ☐ 5 ☐ 6 ☐ | 7 ☐ 8 ☐ 9 ☐ | 10 ☐ 11 ☐ 12 ☐ |
| 2. Enrolled HIV patients who are overdue for care | ...would not be identified or tracked. | …are inconsistently identified and tracked (e.g., if a provider happens to notice that the patient has not received needed care). | ...are systematically identified. But follow-up with such patients is inconsistent and/or limited (e.g. telephone reminder). | ... are systematically identified AND a staff member is consistently assigned the task of following-up with such patients to help re-engage them in care |
| Score | 1 ☐ 2 ☐ 3 ☐ | 4 ☐ 5 ☐ 6 ☐ | 7 ☐ 8 ☐ 9 ☐ | 10 ☐ 11 ☐ 12 ☐ |

| **Components** | **Level D** | **Level C** | **Level B** | **Level A** |
| --- | --- | --- | --- | --- |
| 3. Linking HIV patients to supportive (wraparound) services | ...is not done systematically. | …is limited to providing patients a list of identified resources in an accessible format. | … is accomplished through a designated staff person or resource responsible for connecting patients with resources. | ... is accomplished through active coordination between the health system, support service agencies and patients, and accomplished by a designated staff person. |
| Score | 1 ☐ 2 ☐ 3 ☐ | 4 ☐ 5 ☐ 6 ☐ | 7 ☐ 8 ☐ 9 ☐ | 10 ☐ 11 ☐ 12 ☐ |
| 4. Clinical care management services for high risk HIV patients | ...are not available. | … are provided by external care managers with limited connection to the practice. | ... are provided by external care managers who regularly communicate with the care team. | ... are systematically provided by the care manager functioning as a member of the practice team, regardless of location. |
| Score | 1 ☐ 2 ☐ 3 ☐ | 4 ☐ 5 ☐ 6 ☐ | 7 ☐ 8 ☐ 9 ☐ | 10 ☐ 11 ☐ 12 ☐ |
